# “Sing it, interpret it”: emotion-voice balance in character-construction for musical theater singers

**DOI:** 10.3389/fpsyg.2026.1838236

**Published:** 2026-08-12

**Authors:** Muci Fu

**Affiliations:** New Zealand School of Music, Victoria University of Wellington, Wellington, New Zealand

**Keywords:** emotion, musical theater singers, performance science, self-awareness, voice

## Abstract

This qualitative study explores how experienced musical theater singers understand and manage the balance between emotional expression and vocal stability during character construction. Data source includes systematic observation, semi-structured interviews and field notes with five high-achieving musical theater soloists from the UK and China. The qualitative analysis of the data reveals a dual influence of emotion and voice, which means that emotions may threaten vocal stability through physiological disruptions but also can enhance vocal expressiveness. To address potential emotional outbursts, singers employ two core strategies: (1) using scientifically grounded vocal techniques to develop various character-related vocal qualities without full emotional immersion, and (2) constructing a dual emotional system during rehearsals that distinguishes between personal and character emotions. The study contributes a practice-based framework for balancing emotional authenticity with vocal safety in musical theater performance.

## Introduction

The essence of contemporary musical theater has gradually shifted over the past 10 years from simply singing to a comprehensive performance, including singing, acting, story and dance (Lei, 2011). This shift necessitates that singers attain a synergistic equilibrium in multiple dimensions. Symonds and Millie (2013, p. 68–70) further states that narrative is the primary element of theater and all other elements, such as singing, dance, and dialogue, serve the narrative and convey the theme of the work. In addition, Edwards and Hoch (2018, p. 185) argue that the readjustment of the singing-acting balance has led to a gradual emphasis on character acting over singing in theatrical contexts. Many studies have shown that the expression of the character’s emotions is crucial in character construction. For example, one of the main methods in Stanislavski and Benedetti (2009, p. 16–17) acting system is to mobilize emotional memories to help actors spontaneously “live in the character” at a subconscious level and express the character’s emotions. In addition, Harvard (2013, p. 33, 232–237) proposes that actors can express a character’s emotions through physical movements and different voice timbres (e.g., crying or kneeling to express sadness), thus, voice is an indispensable part of musical theater.

However, during the process of constructing and conveying the character’s emotions, some potential risks may emerge, for example, singers could be strongly affected by their own subjective emotions, which shape complicated feelings (Jürgens et al., 2015, p. 196; Scherer et al., 2010, p. 68–71), that are difficult to control. Furthermore, the more the performer is immersed in the character’s world, the less aware they are of the objective scene and authenticity (Konijn, 2000, p. 93). This creates a dilemma: if singers are being overly emotional may have a physiological impact on singing, for example, producing phlegm, then ruin a performance (Dunbar, 2016, p. 71).

Therefore, singers should observe their emotional state and not sacrifice their vocal stability in order to express the character’s emotion, so as to achieve a balance between emotional expression and vocal stability. However, there is currently little literature on how musical theater singers prevent an imbalance between these two vital aspects in character construction. The aim of this study explore:

(a) How do musical theater singers understand the relationship between emotion and voice in character construction?

(b) What strategies do experienced singers use to balance emotional expression and vocal stability?

This study aims to call on musical theater teachers or singers to pay more attention to the balance of emotions and voices in performances.

## Literature review

### Relationship between voice and emotion in singing

Emotions and voices are closely related. Emotion is acoustically encoded in the voice through systematic variations in acoustic parameters and vocal characteristics, such as fundamental frequency (F0), vibrato, shimmer (amplitude perturbation), tempo, speed, and loudness (Juslin and Laukka, 2003, p. 23–26; Scherer et al., 2017, p. 6–10).^1^ These acoustic features serve as reliable channels for conveying specific emotional intentions, enabling listeners to discern emotions like joy, anger, sadness, and fear even in sung utterances (Chua et al., 2015, October, p. 4; Eyben et al., 2015, p. 2, 5; White, 2016, p. 98). Notably, this coding can also operate effectively independent of lyrical content, as singers can modulate vocal quality, intensity, and vowel articulation [e.g., the open vowel (a)] to convey emotions. This capacity for emotional voice is central to musical theater performance, where voice functions as one of the critical instruments of narrative and character construction.

However, emotions can affect the stability of the singer’s voice, as Konijn (2000, p. 93) proposes that spontaneous emotions that naturally occur in life are variable and unpredictable, and the loss of emotional control can affect muscular engagement during singing, leading to vocal instability (Dunbar, 2016, p. 71). Their views are different from Stanislavski and Benedetti (2009, p. 16–17) and Benedetti (2013, p. 8) acting systems, because they both emphasize the importance of spontaneously “immersing into the character” at a conscious level in character construction through the method of “emotional memory.” However, Stanislavski’s theory of emotional memory has a “danger of emotional outbursts” reputation, such as the negative dissemination of this theory by Stella Adler (Hetzler, 2020, pp. 88–94). Therefore, the singer is caught between the artistic imperative to “live the part” and the physiological imperative to maintain voice control.

### Current approaches to balancing emotion and voice

Current literature proposes solutions to this imbalance from two areas: acting emotions and various vocal qualities based on voice science.

### Acting-focused strategies

The stream of research seeks to refine approaches for accessing and constructing emotions while safeguarding the balance between emotion and voice. Critical reevaluations of Stanislavski’s “emotional memory” highlight its potential for provoking uncontrolled emotional recall, leading to suggestions for maintaining a “safe artistic distance” from personal traumatic material (Evangelatou, 2021, p. 27). Other frameworks offer more structured alternatives. Konijn (2000, p. 58–67) divides acting emotions onstage into task emotions and character emotions, which is a framework that enables emotional regulation by clarifying the type. In her explanation, the character’s emotion is public, an emotional action rather than a real emotion (Konijn, 2000, pp. 79–80). According to Frijda (1986, p. 454) cognitive emotion theory, task emotions originate from the individual’s interaction with the environment, such as the audience or co-performer’s responses (Kawase, 2014, p. 57), etc., and they are functional responses arising from the individual’s concerns in dealing with environmental demands. Konijn (2005, p. 76–80) further demonstrates through experiments that actors experience task emotions based on their own concerns on stage, which are not significantly correlated with the character emotions. This cognitive separation provides a scaffold for emotional regulation, allowing actors to channel task-related arousal without being subsumed by the character’s emotional reality. In addition, there are other supporting approaches for the balance between emotion and voice. For example, the use of mental imagery (e.g., movie scenes, personal memories, etc.) to induce the expected emotions (Aly et al., 2024, p. 382); the integration of mindfulness into acting to improve actors’ focus and psychological resilience (Ingleson, 2022, p. 171–172); and a crosspoint acting system that integrates the Stanislavski acting system and Mary Overlie’s six viewpoints (i.e., space, shape, time, story, etc.) to provide actors with virtual emotional resources, freeing them from dependence on personal emotional memories and thus preventing emotional outbursts and vocal instability (Atkins, 2022, p. 62–64).

### Vocal-technical strategies

Voice science emphasizes developing a versatile vocal technique that can signify emotion through conscious physiological adjustments, thereby reducing dependence on internal emotional states. Research confirms that listeners associate specific vocal qualities, such as breathy voice, sob, falsetto, belt, or twang, with distinct emotional categories, such as sadness, happiness, anger, or fear (Gobl and Chasaide, 2003, p. 17). Estill Voice Training System provides a detailed, anatomically-grounded framework for voluntarily controlling the muscular configurations that produce these different qualities (e.g., larynx position, aryepiglottic narrowing, tilted thyroid cartilage, etc.).^2^ By mastering the physical “recipes” for emotional voices, singers can produce acoustically convincing expressions of sadness, anger, joy, or other emotions through skilled manipulation of the vocal tract shape, breath management, and resonance balance, rather than through sheer emotional surrender (Sundberg, 1987, p. 20–23; Titze and Martin, 1998). Harvard (2013, p. 232–237) suggests applying different voice qualities and their physiological interpretations from the Estill Voice Training System to different emotional expressions in musical theater singing. For example, the quality of sob or belt may be used to express sadness or anger. Thus singers need to make some adjustments to muscular engagement. Therefore, they do not have to immerse themselves in characters.

### Research gap

Despite these distinct scholarly streams, a significant integrative gap remains. Vocal science studies often focus on acoustic outcomes or isolated techniques outside of full performance contexts, while acting theories frequently neglect the unique physiological and acoustic demands of sustained, pitched voice under emotional load. Consequently, there is limited research that captures the synthesizing praxis of the musical theater singer, such as the balance between the imperative for emotional truth and the requirement for vocal health and stability during character construction and live performance. Although Harvard (2013, p. 232–237) provides a valuable applied proposition, its validation through the lived experiences and reflective practice of expert performers is scant. Therefore, this study directly addresses this gap. It adopts a qualitative and performer-centered approach to explore how experienced musical theater singers conceptually understand and manage the balance between emotion and voice.

## Research method

This study adopted a qualitative interpretivist paradigm to elicit the subjective experiences and interpretive perspectives of five musical theater singers on the body and mind engagement to balance emotion and singing in constructing characters in musical theater performance (Cohen et al., 2002; Hennink et al., 2020; Saunders et al., 2015). A multiple case study design was employed to capture the idiosyncratic experiences of each participant while enabling cross-case comparison (Creswell et al., 2007). It aimed to generate contextually grounded and theoretically informed conclusions.

### Participant selection

Yin (2011) suggests that the selection of participants in qualitative research is purposive, aiming to select participants who can provide the richest and most valuable data for the research. Therefore, five high-achieving musical theater singers from the U. K. and China were purposively selected based on three criteria:

(a) ≥ 10 years of cumulative experience in musical theater performance, direction or pedagogy.
(b) A proven track record of portraying numerous classical characters in Chinese (for Chinese participants) or Western musical theater works. For example, having played lead roles in at least five musical theater works; and having at least one of the roles played in the musical theater that is recognized in China (for Chinese participants) or worldwide.
(c) An established professional standing in the field of musical theater, such as holding academic positions in renowned conservatories, having starring characters in West End/Broadway productions, or receiving industry recognition.

This study received ethical approval, and all interviewees agreed to have their real information, including names, workplaces, awards and performances, used in this research. The detailed information of each participant is presented below:

Yizi Yu: A professor (soprano) in musical theater and opera at Wuhan Conservatory of Music in China. With expertise in cross-cultural vocal pedagogy, including training in contemporary music in Canada, she has released multiple solo albums (covering contemporary and classical music) and performed solo recitals across China, North America and Europe.

Jun Yi: He is a professional singer (baritone) who has been working at Hubei Opera and Dance House in China for over 10 years. Since his debut, he has portrayed numerous iconic characters in hundreds of Chinese and Western musical theater and opera. He now specializes in performance for original Chinese small-theater musical theater and Chinese operas. For example, he performed in the immersive musical theater *The Lighthouse*, composed by Frank Wildhorn in Wuhan, playing one of the three “lighthouse keepers.”

Claire Moore: She is a British musical theater soprano and actress. She is best known for her roles as Christine in Andrew Lloyd Webber’s West End production of The Phantom of the Opera and Ellen in Claude-Michel Schönberg’s Miss Saigon. She has been a leading lady in London’s West End for over four decades, appearing in numerous landmark productions and receiving widespread acclaim for her exceptional singing and dramatic flair. For example, she received a Laurence Olivier Award nomination for Best Actress in a musical theater work for her character as Cora in *Girls* (2017 West End production).

Rosie Ashe: She is a distinguished British soprano who trained at the Royal Academy of Music and the Center Opera London. She has played important characters in many musical theater works, such as Carlotta in the original cast of Andrew Lloyd Webber’s musical theater production *The Phantom of the Opera* (Her Majesty’s theater) and Madame Thénardier in Claude-Michel Schönberg’s musical theater work *Les Misérables* (Palace theater).

Duncan Smith: He is a famous and highly respected baritone performing in London’s West End and on Broadway. His performance throughout America, Europe and Australia in productions, such as Max Detweiler in Richard Charles Rodgers’ *The Sound of Music,* which toured the UK and Judge Turpin in *Stephen Sondheim’s Sweeney Todd* on American Broadway. Additionally, as an actor, he has appeared in films, including *Mamma Mia! Here We Go Again*, directed by Ol Parker.

The small sample size is justified by the “typicality” and “information saturation” of the participants: as elite practitioners with diverse cultural, educational and performance backgrounds, their experiences are representative of high-level musical theater practice and the in-depth data collected through extended interviews and multiple observations ensured no new themes emerged, satisfying the requirements of qualitative research validity (Saunders et al., 2015).

### Data collection

Data were collected over 12 months (April 2024–April 2025) during the second year of my doctoral candidature. Data collection encompassed semi-structured interviews, systematic observation of live performance recordings and field notes with triangulation applied to enhance data credibility (Yin, 2009). To provide a clear chronological overview, the data collection process was structured into four phases, as Table 1 shows:

**Table 1:** Chronological overview of data collection.

Period	Activity	Purpose
From January to April 2024	Preparation	Obtain ethical approval; contact interviewees to sign Information Sheet and Consent
From May to September 2024	Semi-structured interview	Collect perspectives on emotion-voice balance and feasible measures
From September 2024 to January 2025	Systematic observation	Observe interviewees’ performance videos and rich data
From May 2024 to January 2025	Field notes	Record supplementary contextual information

### Semi-structured interview

Interviews are regarded as a critical tool for case study (Yin, 2009). Cohen et al. (2007) also emphasize the flexibility of the interview: collecting data through multiple-sensory channels involving verbal (spoken, heard) and non-verbal (felt, seen). To ensure depth and authenticity, interviews were conducted in participants’ first languages (i.e., English and Mandarin). Since none of the interviewees lived in New Zealand, all interviews were conducted via Zoom and Tencent Meeting (a Chinese online meeting app). Each interview was standardized to 60–90 min, with a consistent framework of 12 open-ended questions covering four core dimensions: (a) factors triggering emotional-voice imbalance during performances; (b) participants’ understanding of the relationship between emotion and voice in character construction; (c) strategies for balancing emotional expression and vocal stability; (d) rehearsal methods for integrating body, emotion, and voice. Non-verbal cues (e.g., pauses, emotional fluctuations, gestures) were recorded in real time to supplement verbal data. The detailed information of each interview is presented (Table 2).

**Table 2:** Detailed information of each interview.

Participant	Way of interview	Date (D/M/Y)	Time
Yizi Yu	Tencent meeting	26/5/2024	58:32
Jun Yi	Tencent meeting	10/6/2024	1:14:37
Claire Moore	Zoom	16/7/2024	1:34:15
Rosie Ashe	Zoom	30/7/2024	47:31
Duncan Smith	Zoom	5/9/2024	1:12:08

### Systematic observation

As noted by Chen (2000) and Morgan et al. (2017), observation provides an important supplementary source of evidence in case study research. Therefore, observations were conducted after interviews. For each participant, two video recordings of formal stage performances were selected to ensure the comprehensiveness of observation data, with the following selection criteria:

(a) Recordings from the past 5–10 years to reflect recent performance practices.
(b) Complete performances (not excerpts) to observe emotional-voice dynamics throughout the production.
(c) Performances of different musical theater genres (e.g., classic musical theater, original musical theater) to capture contextual variability.

Observations were conducted using YouTube and Bilibili (a Chinese video platform), with a structured observation checklist to record key indicators, such as emotional expression (e.g., facial expressions, body movements, eye contact with the audience/co-performers); vocal characteristics (e.g., vibrato intensity, timbre changes, pitch stability); coping strategies during emotional arousal (e.g., breathing adjustments). All observations were recorded in text form, with timestamp annotations for cross-referencing with interview data.

### Field notes

Field notes were documented throughout the data collection process (including during and after interviews, and while watching participants’ performance videos) to record supplementary contextual information. The content included:

(a) Participants’ emotional states during interviews (e.g., Jun Yi noticeably spoke faster and used more body language when discussing his performance experiences where his emotions were affected by the audience).
(b) Background details of observed performances (e.g., audience size, venue type, stage setup).
(c) The researcher’s initial reflections on emerging themes (e.g., consistent mentions by Jun Yi, Claire Moore and Duncan Smith of “writing character autobiography”).

These notes helped contextualize interview and observation data, enhancing the depth of subsequent analysis.

### Data analysis

Thematic analysis was adopted for data coding and interpretation, following Braun and Clarke (2006, p. 86–87) six-step framework, such as familiarizing with data, generating initial codes, searching for themes, reviewing themes, defining themes, and writing the report, to ensure systematicity and transparency. First, all interview recordings were transcribed verbatim, and observation notes and field notes were organized chronologically and by participant to form a comprehensive dataset. Then, I coded keywords, sentences, and paragraphs related to the research questions. Coding dimensions were predefined based on the research aims: “the dual influence of emotion and voice,” “balancing strategies,” and “body engagement and a framework of vocal techniques.” Each code was clearly defined to ensure consistency. Subsequently, coded data were compared to identify cross-case similarities and differences. For example, “writing a character autobiography” (Jun Yi) and “establishing a character’s logical system” (Rosie Ashe) were grouped into the theme “balancing strategies.” Cross-case analysis followed a “replication logic,” which means common themes across all participants were identified as generalizable strategies, while unique themes were analyzed in relation to participants’ cultural backgrounds or performance contexts. Finally, all themes were reviewed to clarify their connections and relevance to the research questions. The final coding focused on two core thematic clusters, including “singers’ understandings of emotion and voice in character construction” and “effective balancing strategies,” forming a logically coherent research findings report.

In addition, to ensure the trustworthiness of this study, three strategies were also adopted. First, I conducted triangulation on data from interviews, observations, and field notes. For example, I confirmed the effectiveness of “abdominal breathing” as a strategy by analyzing Duncan Smith’s interview descriptions and observing breathing patterns in performance videos. Secondly, it includes the researcher’s self-reflection as a singer with many years of experience in performing classical and musical theater. Additionally, the researcher’s supervisor was invited to participate in the coding reliability check and the entire analysis. Finally, participant review. Key findings relevant to each participant were shared via email (if they wished to read) for verification and revision, thereby ensuring the accuracy of data interpretation.

## Findings

All participants were experienced musical theater singers or professors. Their answers to the interview questions were based on their experience and understanding of musical theater character construction and singing. The following two sections elaborate on the answers to the two research questions of this study, namely the relationship between emotions and voices in musical theater character construction and effective balancing strategies.

Research Question 1: Understanding the Emotion-Voice Relationship.

### Dual impact of emotional engagement on voice

The interviews showed that all singers acknowledged that emotions can either overpower or enhance the vocal expressiveness in character construction. All interviewed singers reported experiencing “emotional crisis” in singing because of some factors when constructing characters. Yizi Yu, Jun Yi, and Duncan Smith both mentioned that the audience and singing partners can influence a singer’s emotions, then affect the singing. For example, Jun Yi said:

When I was performing in a musical theatre work, ***, I played the character of ***. That was my last show for this work, and so many fans came to watch my performance. When I went on stage to sing an aria, many audience members were already crying. They probably thought that I would leave after this performance and would not perform this work again. They were very emotional…When I was speaking the dialogue, my tears started to flow. When I sang the first line, my voice was choked…

His experience vividly illustrated how the audience’s emotional responses affected him, making him transcend character-self boundaries, triggering personal emotional fluctuations and disrupting vocal stability. As he said, “even with my extensive stage experience, I have to admit that this emotional connection was overwhelming.” He noted that this effect was particularly pronounced for younger singers. He also described the experience of one of his young partners when singing their trio in this song:

*We had a trio, and one of the singers was younger. He was so moved by this scene that he lost control of his emotions on stage. What was the final result? Because of sobbing, his voice was full of phlegm, he could no longer express his voice and acting, and he finished the performance twitching from beginning to end. Fortunately, I was the main melody, so the final effect was not too bad…*

His descriptions illustrated that the lack of emotional control directly led to a physiological obstacle for this young singer in singing. These physiological reactions, such as “sobbing,” “full of phlegm,” and “twitching,” caused tension in the laryngeal muscles, and phlegm affected the closure of the vocal folds, directly leading to difficulty in voice production. According to him, at that time, this young singer was unable to complete the whole song due to a lack of emotional control. Yizi Yu supported his view, and she said:

*Contemporary Chinese small-theatre musical theatre…They offer a zero-distance connection with the audience, creating an immersive performance. This viewing experience is novel and participatory. However, performers and audience are also more likely to influence each other.*

According to her, small-theater musical theater refers to musical theater that is smaller in scale than traditional musical theater, including a smaller stage, fewer seats, simpler stage setups, and lower costs. In this type of production, the boundaries between the stage and the audience were blurred. Therefore, the audience’s reaction may have a magnified impact on the singers’ emotions. Duncan Smith affirmed the Yizi Yu and Jun Yi’s point of view, further stating that, similar to how the audience’s emotions affect the singer, the collaboration between the singer and their singing partners can also influence the singer’s emotional state. He said:

*I played a general in a Hollywood film. At the end of the film and at the end of the show, all his old army buddies surprised him on stage. He had to make a speech, and I thought, and I know that he had to cry…I do not know where it came from, but it was the thoughts.*

These external factors have a clear impact on the singer’s emotions. Jun Yi, Claire Moore, Rosie Ashe, and Duncan Smith, on the other hand, also emphasized that some internal emotional factors, such as the use of emotional memory, may also lead to an imbalance between emotion and voice in character construction. Jun Yi further explained the use of emotional memory: selecting matched (characters) emotions from the singer’s experienced emotional “database” to construct characters. Claire Moore and Rosie Ashe reported their point of view on what they considered the negative side of the use of emotional memories:

*Claire Moore: If the happiness or sadness, or other emotions induced in a singer onstage (regardless of any approaches used) were not consciously controlled, it may lead to excessive emotional expression, then interrupt singing rather than enhancing emotions. Frequent and unregulated crying or laughing during the dialogue may make the audience feel unreal and distract their attention from the story.*

*Rosie Ashe: If I have to sing something about heartbreak, I use emotions like an actor. An ex-boyfriend of mine came to my show, and he said, I cried when you sang that song. I said that was about you. I used him. I was talking about loving someone, and it worked. I remember I used a memory of a broken heart, and I was already very sad at the time because I used it…*

Rosie Ashe’s admission of feeling “very sad and heartbreaking” confirmed that her own emotions were truly affected by the character’s emotions. Although her experience proved that using personal emotional memory was an effective approach to awaken emotions similar to those of the character, constructing the character’s emotions based on past experiences was not only prone to falling into an “emotional vortex,” but long-term use may also bring the risk of mental problems. Therefore, she admitted that singers had to use their own emotional experiences with great care when interpreting characters to avoid affecting their voices. Jun Yi echoed this view, and he said, “The richer a singer’s emotional experience was, the more he/she can match the most appropriate emotions of the characters from the emotional library, but it also means that this depth made it easier to fall into emotional dilemmas.” In addition to this situation, Claire Moore mentioned the negative impact of the singer’s personal emotions on constructing the character’s emotions. She explained that the personal emotions specifically referred to the private emotions stemming from the singer’s private life or events, which were unrelated to the characters. The difference between this and experienced emotions lies mainly in the relevance to the characters. Rosie Ashe also criticized the tendency that some singers to bring their own negative emotions into their characters, emphasizing “the essence of the audience’s expectation of the performance was escapism rather than bearing the singer’s own emotions.”

Jun Yi, Claire Moore, Rosie Ashe and Duncan Smith both explicitly criticized the behavior of interrupting the music and story due to the lack of emotional control. After all, this was unfair to the director, writers, first-time audience, and other singers onstage. As Jun Yi described,

*I noticed the confusion and helplessness of some audience members, maybe they are new, when the stage was in chaos.*

However, all singers acknowledged that all these factors are not entirely harmful, and they can also enhance a singer’s vocal expressiveness. For example, positive feedback and cooperation from the audience can greatly encourage singers and help them with their singing and character development. Yizi Yu and Jun Yi answered:

*Yizi Yu: When I attended the musical theatre Sleep No More, they did not sing, and it was more like an immersive theatrical performance. For example, there was a room where, like performance art, an actor was frantically dancing in front of a mirror. Suddenly, he walked up to me and handed me a note, and everyone gathered around, wanting to see what was written on it… This kind of acting, where the singer and audience interacted seamlessly, was incredibly exciting and novel…*

Jun Yi: Actors and audiences should complement each other, moving one another. Once, I played the role of P**, a truly villainous character. Someone in the audience got very emotional and shouted, “Down with P**! P** is a bad guy!” This audience member was completely immersed in my performance, identifying with the character and the actor himself. This was a huge encouragement for me, allowing me to be more engaged and relaxed in my subsequent performances.

In addition, Jun Yi, Claire Moore, Rosie Ashe, and Duncan Smith, based on their own experiences, all indicated a clear consensus on the value of emotional memory in character construction and that the careful use of emotional memories and personal emotions can help to construct a charming performance. For example, Jun Yi and Duncan Smith said:

*Jun Yi: Rich life experiences and emotional experiences are of great help to my stage performances. They can deepen the tension in my music and acting.*

*Duncan Smith: I would say feelings. So, it does not matter if [you] feel sad, happy, angry or nasty. It’s there, from your life. And I think that if you do a long ‘run’, you learn the technique of being able to tap into these emotions for the journey.*

Rosie Ashe agreed with their views. Based on her performance experience above, she made full use of the emotional memory, a “heartbreak emotion,” in constructing the character and achieved good results. However, she also expressed that although she genuinely felt the emotion of “heartbreak,” she remained keenly aware that she was constructing the character rather than experiencing it in reality. She said:

*I used the emotion of heartbreak, but it came from memory. I did not really bring it on stage. You should leave it at the stage door.*

From her descriptions, such as “used the emotion,” and “leave it at the door,” suggested that while memories or experiences of “heartbreak” can serve as emotional materials to fuel the authenticity of a character’s construction, they should not allow the emotions to overwhelm the singer or drag these emotions back into real life. Claire Moore focused on the positive impact of the singer’s personal emotions on character construction. Although Claire Moore stated that using the characters as a medium to vent the singer’s personal emotions was not advisable, as it may cause the audience to be pulled away from the characters and harm the singer’s mental well-being, she also noted a nuanced exception, that a singer’s personal emotion may enhance the emotional power of the character and give the voice more color, particularly for the audience familiar with the singer’s background. She said:

*One of my students is a fine example of a woman who would come on stage whose life was a come in complete mess. She was in complete chaos. Yet she never gave anything away on stage. She sang her songs, and she did not break down in tears in musical works…For the audience. I just think that it is so touching that she would hold onto it, and her voice seemed to be even more moving…*

In sum, all five singers reported that emotional engagement was necessary in character construction, but it must be kept within a range that does not affect vocal stability. External factors have a two-fold impact on singers’ emotions, as they can either help create an expressive character or lead to a failed performance.

Research Question 2: Strategies for Balancing Emotion and Voice.

### Voice technique serves as a “safe framework” for emotional expression

The interviews showed that all interviewed singers regarded vocal technique as a guarantee for emotional expression. For example, Claire Moore and Jun Yi pointed out:

*Claire Moore: Only with vocal technical support could one ensure the freedom of the singer’s vocal emotions.*

*Jun Yi: Daily emotional voices, such as angrily shouting, though common in real life, were inappropriate on stage, as they risked undermining the singing and may even cause vocal fold injury.*

Therefore, an immature vocal technique may limit the emotional expressions. As a vocal teacher, Yizi Yu said, “Vocal technical errors may destabilize emotions.” She observed that when young singers made some technical mistakes on stage, their emotions apparently fluctuated, manifesting in uncontrolled tension, which was visible in their body movements, eyes, acting and facial expressions. For example, the voice began to tremble, the eyes wandered, and the personal expression revealed “discomfort” rather than the character’s emotions. In this state, the expression of the character’s emotions was greatly diminished.

Therefore, all participants emphasized that musical theater singers needed to convey character emotions by changing different timbres, based on scientific vocal techniques. Drawing on participants’ perspectives, singers do not have to fully immerse themselves in the character’s emotions, as they can convey those emotions through their emotional voice. Specifically, the data revealed that different emotions might be controlled through the control of breathing and vocal muscles. For example, Duncan Smith highlighted the foundational role of breathing in this process. He advocated abdominal breathing^3^, arguing that chest breathing may cause muscle tension, thereby causing vocal tension. As he said,

*If you watch good singers, they all breathe from the diaphragm…However, by adjusting breathing frequency and pattern, singers can construct distinct emotional states. For example, rapid chest rise from intense breathing may signal anxiety or anger, while core muscle engagement could infuse the singing with power.*

Rosie Ashe agreed with Duncan Smith’s view that abdominal breathing is the priority, emphasizing that the breathing exercise and text work laid the foundation of emotional expressions. She also suggested that modifying the text rhythm within the breathing cycle was critical to conveying emotions.

In addition to the breathing cycle in singing, Claire Moore noted the importance of character voice in musical theater character construction. As she said, “Only a small portion of musical theater notes, perhaps even less than 5%, risks harming singers.” This meant most of the time, singers were able to change timbre or vocal quality related to characters to interpret them and convey emotions freely. As she explained:

*I may reduce the support of core muscles in some singing sections and add some “cracks and creaks” to the voice. This not only meets the musical theatre’s requirement for the “truth” of the character but also does not harm my voice…*

The voice, especially for musical theater singers, was highly malleable, requiring timbres that aligned with the character and different emotions for both dialogue and singing. She added, “For example, a bright and resonant twang voice for Disney-style characters, a sob voice for crying scenes, a powerful belt voice for wild or excited moments, and so on.” However, all singers emphasized that the transformation of vocal qualities must be based on scientific vocal technique. The content the interviewed singers provided was consistent with their performances in observed videos. For example, in Claire Moore’s performance video, “All I Ask of You” from *Phantom of the Opera.* She played Christine, performing this piece with her partner, the character Raoul. In response to Raoul’s intense love, she sang very softly with a breathy sound.^4^ In the lower register, she tended to open her mouth horizontally, and she described that she also slightly lowered her soft palate to achieve a more natural voice, which was also conducive to showing the character’s emotions. Although she said that a speaking voice in the lower register slightly decreased the engagement of core muscles compared to singing in the higher registers, this timbre fully transferred love, hope and passion. Meanwhile, Claire Moore’s body movement matched her timbre. For example, as the song progressed emotionally, they embraced, and their eyes constantly locked. In Yizi Yu, Jun Yi and Rosie Ashe’s performing videos, they all tried to authentically interpret the characters with a natural speaking or belting voice. This vocal quality facilitated their ability to convey a variety of emotions, as listeners can intuitively sense the emotions they convey through changes in speed, timbre, rhythm, and other characteristics. Jun Yi said:

*I prefer to use a more belting voice to express some tense emotions, such as anger and anxiety. However, this emotional voice has a limited vocal register for me and is usually concentrated before the passaggio.*

However, as he cautioned, using a speaking/belt voice in the higher register may put more pressure on the vocal folds. He described the physicality of this voice production,

*My vocal folds suddenly closed, with the core muscles, the waist and back muscles engaging explosively to produce a roar-like and powerful sound.*

Therefore, all interviewed singers also emphasized maintaining a relatively classical timbre in singing high notes, which may help to relieve the pressure on vocal folds, express emotions, and strengthen the emotional signals of the sounds.

In sum, all the participants emphasized the importance of using scientifically based vocal techniques to transform different vocal qualities in conveying a character’s emotions. Therefore, singers do not need to fully immerse themselves in the character’s emotions, and they can convey those emotions through their own emotional voice. This effectively reduces the risk of singers losing emotional control while constructing a character, while still ensuring the integrity of the character.

### Rehearsal: dual emotion-awareness in character construction

In response to the potential risks of imbalance between emotion and voice in the character construction in musical theater, the interviewed participants provided some suggestions based on their experiences and found them to be effective in their decades of performing careers. Jun Yi, Claire Moore, Rosie Ashe and Duncan Smith both mentioned the function of rehearsal to connect the body, character and emotion. For example, Claire Moore and Duncan Smith emphasized that rehearsals provided a safe space to release their real emotions without reservation, allowing thorough exploration of the character’s emotions. As Claire Moore said:

*I think if you can access those emotions in rehearsal, getting out of your system, so you know what the emotion is you are trying to convey, then you have to not fake it, but you have to look after the voice first during performances…*

The goal here was twofold: first, to allow the singer’s body to establish a connection with the character’s emotions and remember how feelings map to vocal expressions, including singing and dialogue; second, to seek the best state of emotional and vocal expression. In rehearsals, singers need not fear performance disruptions from uncontrolled emotions, enabling them to repeatedly refine the integration of the character’s emotions with their bodies. Through emotion-singing trial and error, for example, testing which degree of sadness or other emotions best balances emotional expressions and vocal stability, singers can find an optimal, balanced mode of expression.

In addition, Jun Yi and Rosie Ashe focused on establishing characters’ logic and constructing the characters’ emotional system during the rehearsal, including appropriate emotions, emotional intensity, and body reactions to emotional expression. For example, Jun Yi elaborated on how rehearsals helped bridge the gap between the singer and the character, especially for less experienced singers:

*For me, when I first come into contact with the script, I feel that the character is far away from me, but now, first, I have the script, I usually write an autobiography for the character. For example: What do you do? What things have you done? How do you treat others?. You need to have continuous thinking that conforms to the logic of the character, so that you can subtly tell yourself what kind of character you are interpreting.*

This autobiographical approach served a key purpose, which was to build the “trust” in the singer’s mind. By constructing a detailed, logical framework for the character, including their background and personality, singers were able to gain greater control over the characters’ emotional construction. Rosie Ashe agreed with this view, she said:

*I believe that it is essential to establish a character’s logical system during rehearsals, including who I am or where I am. This clarity allowed singers to select the vocal quality that suits the character, including what voice should be used when expressing emotions. For example, how a withdrawn person expressed joy or anger would be different from a bold and free-spirited person, with changes in timbre, muscular engagement and actions.*

This process of aligning the vocal quality and character unfolded before and during rehearsals. Jun Yi and Rosie Ashe also stated that once a character construction system, including voice, emotions, body movements, makeup, etc., is established, it can effectively help singers distinguish between character emotions and personal emotions. In interviews, Jun Yi, Claire Moore, Rosie Ashe and Duncan Smith both indicated that singers needed to clarify the difference between their own emotions and the character’s emotions in character construction. They shared their opinions:

*Jun Yi: The emotional unity between the singer and the character is only an ideal state, which is very hard to achieve, but the singer cannot be swallowed by emotions.*

*Claire Moore: Emotions came from deep inside and established a connection with the body, so the singer did not require excessive displays, like constant crying, to convey.*

*Rosie Ashe: Personal emotions are private, but the character’s emotions are public.*

Through imagination or induced emotional memories, they may have genuinely felt the emotions of the characters or matched the emotions of the corresponding experiences. Yet crucially, all interviewees affirmed that rationality remained dominant, at least during the performance, singers need to pay attention to much more than just character construction during a performance, including voice, body movements, and coordination with the band, partner or pianists. Even Duncan Smith, who emphasized the need to “believe in the story and the characters from their hearts,” acknowledged that the characters were ultimately fictional, expressed by singers.

## Discussion

The findings show that the impact of emotion on vocal stability is twofold. Most participants (Jun Yi, Claire Moore, Rosie Ashe and Duncan Smith) acknowledged experiencing a “vocal crisis” triggered by emotional fluctuations. In particular, the adverse physiological reactions caused by emotions (Jun Yi). This finding is consistent with Dunbar (2016, p. 71) view that uncontrolled emotions can directly disrupt vocal stability through physiological responses such as laryngeal muscle tension and increased secretions. It is also noteworthy that the chain reactions described by Jun Yi and Duncan Smith in this study, triggered by audience or partners’ feedback (e.g., crying), can be understood using Frijda (1986, p. 454) cognitive-emotion theory: singers subconsciously assess these external events as “concern” related to their own performance success, thus triggering real, sometimes intense, emotional and physiological responses. However, experienced performers may develop more complex assessment and regulation mechanisms than young singers.

The findings further suggest that emotional impact stems not only from external interactions (e.g., audiences and partners) but also from internal processes, particularly the use of Stanislavski and Benedetti (2009, p. 16–17) “emotional memory” method. For example, the use of emotional memory of Rosie Ashe and Jun Yi’s experience confirmed (Hetzler, 2020, p. 94) perspectives that emotional memory remains effective in character construction, but also highlighted the potential risk that actors may fall into an emotional “vortex,” blurring the boundaries between the character and themselves. Excessive immersion may lead to a diminished awareness of the objective performance scene and then affect the vocal stability (Konijn, 2000, p. 93).

However, based on the participants, the same emotional sources can also enhance the authenticity and impact of a performance. All participants agreed that the prudent use of emotional memories and personal experience can deepen character construction. Crucially, the findings emphasized the “use” of these emotions rather than “immersion” in them. This description resonates with Konijn (2000, p. 58–67) distinction between “task emotions” and “character emotions.” Participants’ experience suggests that successful performance does not involve completely eliminating personal, genuine emotional responses (task emotions), but rather distinguishing them from the “character emotions” that need to be publicly constructed through conscious awareness, using the former as a source of material to inspire the latter rather than an uncontrollable source. This not only addresses the debate surrounding the risks of Stanislavski’s emotional memory method (Hetzler, 2020) but also points to the necessity of maintaining a “safe artistic distance” (Evangelatou, 2021, p. 27).

The findings indicate that effective solutions for the balance between emotion and voice can be divided into two aspects: transformation of vocal quality and rehearsal. All participants emphasized that mature vocal technique is a prerequisite for allowing for the safe expression of emotion and voice. The findings suggest that singers do not always need complete emotional immersion to convey a character’s emotions. Instead, they can “create” an emotional voice through conscious, physiologically based technical adjustments. For example, participants detailed how they match characters and situations through breath control (e.g., adjusting the frequency and pattern of abdominal breathing and chest breathing), the shape of vocal tract adjustments (e.g., altering mouth shape and soft palate position to produce a breathy or more natural timbre), and the use of specific vocal qualities (e.g., a speaking voice, a crying sound, and a belt voice) (Jun Yi, Claire Moore, and Duncan Smith). The changes of vocal qualities mentioned in the findings align with Sundberg (1987, p. 20–23) theory of voice science, which states that vocal tract shape determines formants, thus affecting timbre, and the participants’ descriptions are examples of shaping specific emotional vocal qualities by altering the shape of length of the vocal tract. The findings also provide practitioner-based evidence for Harvard (2013, p. 232–237) view linking vocal quality with emotional expression in the Estill Voice Training System.^5^

The findings also show that the rehearsal process can be regarded as a crucial space for integrating body, emotion, and voice, and for establishing a “dual awareness between self and character.” Participants (Jun Yi, Claire Moore, Rosie Ashe and Duncan Smith) emphasized that rehearsal is a safe experimental space for exploring the relationship between emotion and voice: singers can repeatedly experiment with how to recall emotional memories (Rosie Ashe), construct character backstories (Jun Yi), and test vocal qualities under different emotional intensities until they find a balance that is both authentic and stable. This process helps singers clearly distinguish between the public and performative “character emotions” and private “task emotions” (Konijn, 2000, p. 58–67). The findings further illustrate that through the rational character logic and bodily memory established during rehearsals, singers can maintain a dominant “task awareness” in performance, which focuses on singing technique, cooperating with their partner, etc., thereby maintaining a controllable “observational distance” from the engaged character emotions, avoiding being overwhelmed by them.

**Figure 1:**
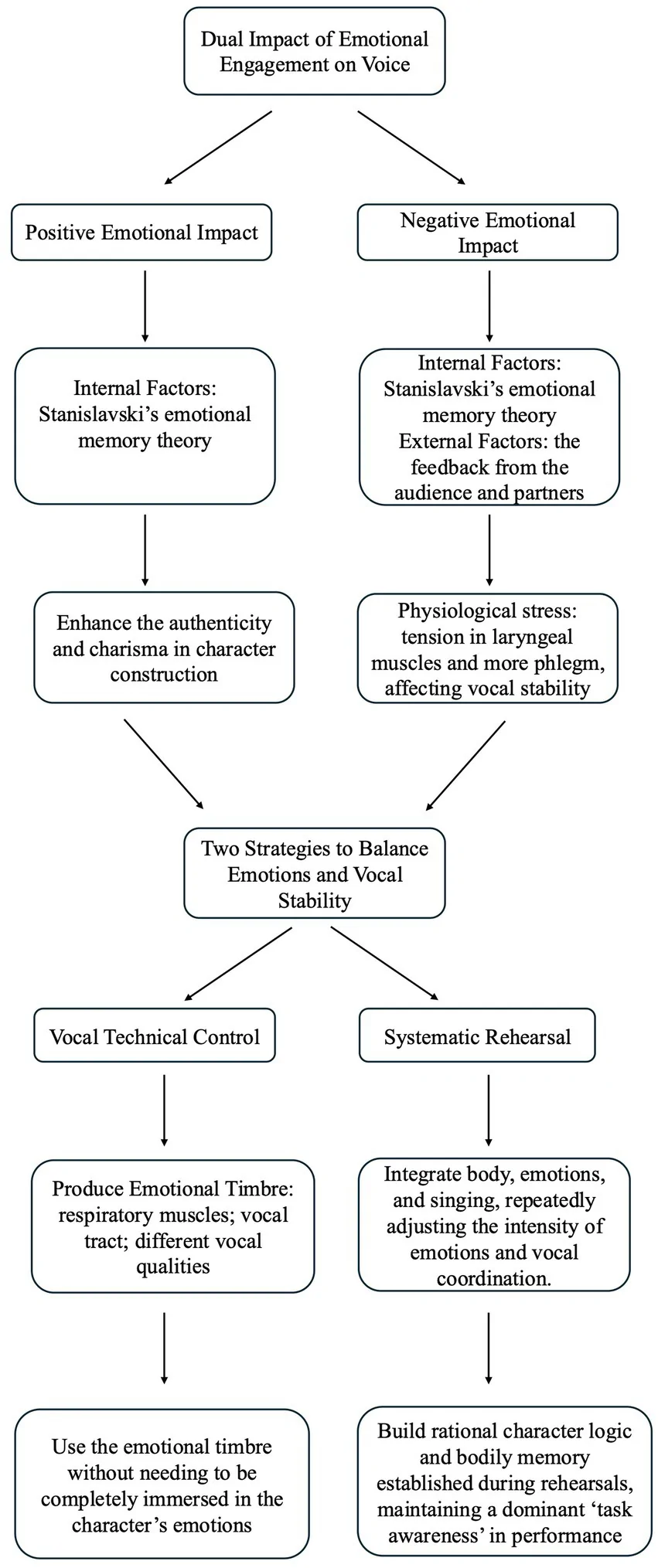
Summary of the core conclusions from interviewees.

Therefore, based on the analysis of findings, the core conclusions obtained from the interviewees are summarized in (Figure 1).

## Conclusion

The study provides a theoretical and practical basis for the transformation of musical theater performance training from “experience accumulation” to “scientific regulation.” It reveals that the balance between emotion and voice for musical theater singers is not a binary choice of “suppressing emotions for singing” or “indulging emotions for characters.” Instead, it is a dynamic balance achieved through three steps: rational understanding of the relationship between emotion and voice in character construction, systematic vocal technical training, and rehearsal preparation. Notably, this finding is different from Harvard (2013, p. 26) view that self-awareness was the denial of self during performance. Rather, this study identifies self-awareness as a clear understanding that transcends singers’ characters, and “taking self-awareness as an anchor” as the core of this balance: neither being swallowed by the character’s emotions nor closing off emotional expression due to vocal technical safety.

However, this study has limitations. As a qualitative inquiry based on a multiple-case study design, the findings are contextually rich but not statistically generalizable. The small, purposive sample of five high-achieving singers, while providing depth and saturation, may not fully represent the broader population of musical theater singers, particularly those with less experience or from different training traditions.

In future research, the case studies can be expanded in both number and scope, including singers at different career stages (e.g., students, mid-career performers) and from diverse cultural and pedagogical backgrounds may enhance the transferability of the findings and allow for comparative analyses of training impacts. In addition, longitudinal research can be conducted to track singers’ development of emotion, voice balance over time, particularly in relation to specific rehearsal methodologies or vocal techniques.

## Data Availability

The datasets presented in this article are not readily available because The Human Ethics Committee at Victoria University of Wellington has requested that the raw data not be made public. Requests to access the datasets should be directed to Muci Fu, fumuci10797@gmail.com.

## Footnotes

1 Shimmer refers to the degree of short-term fluctuation in the amplitude of sound.

2 Estill Voice Training System (Level Two) Workbook, written by Mary McDonald Klimek with Kerrie Obert and Kimberly Steinhauer. (Published by Estill Voice Training System International, LLC, 2005). This workbook is published for use in the Estill Voice Training System courses and presents the timbre characteristics and physiological explanation for some vocal qualities (e.g., bel canto, belt, speech, sob, falsetto, twang, etc).

3 The abdominal breathing means that when inhalation, the lungs expand, which makes the diaphragm flatten and then causes the abdominal muscles to expand. Chest breathing means that when inhalation, the chest cavity rises, causing the diaphragm to rise and the abdominal muscles to contract.

4 Breathy voice means the vocal folds do not close completely during the voice production.

5 Estill Voice Training System (Level Two) Workbook, written by Mary McDonald Klimek with Kerrie Obert and Kimberly Steinhauer. (Published by Estill Voice Training System International, LLC, 2005). This workbook is published for use in the Estill Voice Training System courses and presents the timbre characteristics and physiological explanation for some vocal qualities (e.g., bel canto, belt, speech, sob, falsetto, twang, etc).
